# Diagnostic Challenges of Functional Cognitive Disorders

**DOI:** 10.1192/j.eurpsy.2023.1947

**Published:** 2023-07-19

**Authors:** J. Petta, A. L. Falcão, G. Soares, A. Lourenço

**Affiliations:** Centro Hospitalar Psiquiátrico de Lisboa, Lisboa, Portugal

## Abstract

**Introduction:**

Functional cognitive disorders (FCD) denotes a complaint about memory function or, less commonly, another cognitive process, in the absence of relevant neuropathology and with evidence of inconsistency between symptoms reported and signs identified at assessment.

Increasing numbers of people with FCD are being identified.

Most are discharged back to primary care without a diagnosis or are given the label of mild cognitive impairment, which is not a synonym for FCD.

**Objectives:**

There is a multitude of terms in the clinic and in the research to describe this kind of complaints. Some terms seem to minimize and normalize the state, whereas others posit an underlying cause.

Given this lack of order, it is a challenge to diagnose it and give it the proper clinical guidance.

This literature-based review aims to fill this gap.

**Methods:**

Data was obtained through an internet-based literature search, using the databases PubMed, Cochrane Library and NCBI. The World Health Organization was also utilized. Nine articles from the last two years were included.

**Results:**

It is listed a nosology in six categories and a selection of clinical features which may help with the discrimination of functional and neurological disease causes of memory disorders.

**Conclusions:**

Patients presenting with complaints about memory function require standard psychiatric and neurological history and examination.

It should be emphasized that these conditions are not diagnoses of exclusion but have positive symptoms and signs that should become well-known.

Nevertheless, we remain uncertain about prognosis and treatments, both psychological and pharmacological. Its development would reduce the burden of patients in the healthcare systems.

**Disclosure of Interest:**

None Declared

